# MiR‐26a‐tetrahedral framework nucleic acids mediated osteogenesis of adipose‐derived mesenchymal stem cells

**DOI:** 10.1111/cpr.13272

**Published:** 2022-06-05

**Authors:** Xiaoru Shao, Zhong Hu, Yuxi Zhan, Wenjuan Ma, Li Quan, Yunfeng Lin

**Affiliations:** ^1^ Department of Stomatology Affiliated Hospital of Jining Medical University Jining Shandong China; ^2^ College of TCM, Shandong University of Traditional Chinese Medicine Jinan Shandong China; ^3^ State Key Laboratory of Oral Diseases, National Clinical Research Center for Oral Diseases, West China Hospital of Stomatology Sichuan University Chengdu Sichuan China; ^4^ Business College China West Normal University Nanchong Sichuan China; ^5^ Sichuan Inspection and Testing Center for Dental Devices and Materials Ziyang Sichuan China

## Abstract

**Objectives:**

Delivery systems that provide time and space control have a good application prospect in tissue regeneration applications, as they can effectively improve the process of wound healing and tissue repair. In our experiments, we constructed a novel micro‐RNA delivery system by linking framework nucleic acid nanomaterials to micro‐RNAs to promote osteogenic differentiation of mesenchymal stem cells.

**Materials and Methods:**

To verify the successful preparation of tFNAs–miR‐26a, the size of tFNAs–miR‐26a were observed by non‐denaturing polyacrylamide gel electrophoresis and dynamic light scattering techniques. The expression of osteogenic differentiation‐related genes and proteins was investigated by confocal microscope, PCR and western blot to detect the impact of tFNAs–miR‐26a on ADSCs. And finally, Wnt/β‐catenin signaling pathway related proteins and genes were detected by confocal microscope, PCR and western blot to study the relevant mechanism.

**Results:**

By adding this novel complex, the osteogenic differentiation ability of mesenchymal stem cells was significantly improved, and the expression of alkaline phosphatase (ALP) on the surface of the cell membrane and the formation of calcium nodules in mesenchymal stem cells were significantly increased on days 7 and 14 of induction of osteogenic differentiation, respectively. Gene and protein expression levels of ALP (an early marker associated with osteogenic differentiation), RUNX2 (a metaphase marker), and OPN (a late marker) were significantly increased. We also studied the relevant mechanism of action and found that the novel nucleic acid complex promoted osteogenic differentiation of mesenchymal stem cells by activating the canonical Wnt signaling pathway.

**Conclusions:**

This study may provide a new research direction for the application of novel nucleic acid nanomaterials in bone tissue regeneration.

## Graphical abstract

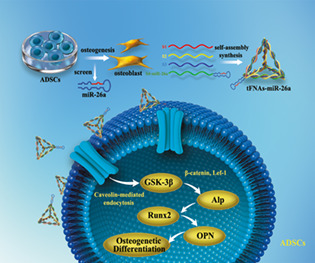

## 1 INTRODUCTION

Micro‐RNAs (miRNAs) attract substantial research attention given that they play important roles in cell differentiation, biological development, and disease pathogenesis.^1^ Further researches use advanced high‐throughput technology like miRNA microarrays to study the relationship between miRNAs and disease, and conduct in‐depth discussions on the mechanism of miRNAs, making miRNAs expected to become new and promising targets in tissue engineering research.^2^, ^3^ miRNAs are a class of small noncoding RNA sequences that act as efficient molecular managers, regulating multiple endogenous processes simultaneously. miRNAs can regulate protein‐coding gene expression, translation process and various biological behaviors of normal or inflammatory cells at multiple levels.^4^ The combined application of mesenchymal stem cells (MSCs) and miRNAs capable of promoting their osteogenic differentiation to repair damaged bone tissue has become a popular field of bone tissue engineering research.^5^, ^6^ According to reports in the literature, miRNA26a plays an active role in osteogenic differentiation of MSCs, and it can enhance the expression of ALP, an early marker of osteogenic differentiation of MSCs.^7^, ^8^ However, due to the single‐stranded RNA instability of miRNA, it is still difficult to use it widely. Thus, screening for a good vector capable of delivering miRNAs is one of the important problems that limit the application of miRNAs in tissue engineering.^9^ Therefore, in this study, we attempt to use novel nucleic acid nanocarriers to promote the transport and stability of miRNAs in organisms, improve the clinical relevance of miRNA therapy, and provide new research methods for bone tissue regeneration.

The so‐called bone defect is the loss of bone caused by trauma, surgery, inflammatory erosion, or various bone diseases and tumors, which results in functional damage to the human body.^10^, ^11^ How to repair bone tissue defects and restore the related functions has become the research focus of clinical doctors. The current method to repair bone tissue defects is bone grafting, which includes autologous bone grafting and allogeneic bone allografting. However, when the length of a bone defect reaches 1.5 times of the diameter of the shaft, it exceeds the critical size of autologous repair, thereby resulting in bone resorption and nonunion.^12^, ^13^ Therefore, how to use tissue engineering technology to repair bone tissue defects becomes the focus of surgeons.^14^, ^15^ Tissue engineering technology mainly includes seed cells, scaffold materials, and other stimulating factors. MSCs, an important member of the stem cell family, are mainly derived from the early mesoderm and a small part of the ectoderm.^16^, ^17^ Significant research efforts have been undertaken in the last decade to develop specific cell‐based therapies and, in particular, multipotent MSCs hold great promise toward such regenerative strategies.^18^ The adipose tissue‐derived mesenchymal stem cells (ADSCs) have the following advantages: easy separation and purification; easy to culture and fast amplification; easy for the patient to accept; and the damage and pain to the donor are limited.^19^ Therefore, this study explored the combined application of new nucleic acid nanomaterials and miRNA to promote osteogenic differentiation of ADSCs and ultimately promote the repair of bone tissue defects.

Tetrahedral frame nucleic acid (tFNAs) is a new type of nucleic acid nanomaterials, which is self‐assembled through strict base pairing.^20^, ^21^, ^22^ Compared with other nanocarriers, tFNAs has good biocompatibility, biodegradability, and biosafety.^23^, ^24^ Compared with single‐strand DNA, which needs the assistance of other carriers to enter the cell, tFNAs can enter the cell through the formed three‐dimensional structure, and it can achieve lysosomal escape and targeted localization by connecting specific nucleic acid sequences.^25^, ^26^, ^27^ In recent years, an increasing number of researchers have applied tFNAs to tissue regeneration engineering to explore its multiple effects on cell biological behavior. It has been found that tFNAs has certain effects on cell proliferation, differentiation, migration, apoptosis, and other biological processes.^28^, ^29^, ^30^ In this study, we attempt to use tFNAs as a carrier to deliver miRNA into cells, and synergistically promote osteogenic differentiation of ADSCs, so as to provide new research ideas and methods for bone tissue regeneration.

## 2 MATERIALS AND METHODS

### 2.1 Materials

A predesigned nucleic acid single strand was obtained from Takara (Dalian, China), and the miRNA was ligated to the nucleic acid single strand at the design stage (Table 1). Antibodies were purchased from Abcam (Cambridge, UK); fetal calf serum, double antibody, and medium were purchased from HyClone (London, USA); osteogenic induction medium was purchased from Cyagen (California, USA); and an alkaline phosphatase assay kit was purchased from Beyotime (Shanghai, China). Other reagents used during the experiment were of analytical grade or better.

**TABLE 1:** Base sequence of each single‐stranded DNA

S1: 5′‐ATTTATCACCCGCCATAGTAGACGTATCACCAGGCAGTTGAGACGAACATTCCTAAGTCTGAA‐3′
S2: 5′‐ACATGCGAGGGTCCAATACCGACGATTACAGCTTGCTACACGATTCAGACTTAGGAATGTTCG‐3′
S3: 5′‐ACTACTATGGCGGGTGATAAAACGTGTAGCAAGCTGTAATCGACGGGAAGAGCATGCCCATCC‐3′
S4: 5′‐ACGGTATTGGACCCTCGCATGACTCAACTGCCTGGTGATACGAGGATGGGCATGCTCTTCCCG‐3′
S4‐miR‐26a: 5′‐UUCAAGUAAUCCAGGAUAGGCUTTTTACGGTATTGGACCCTCGCATGACTCAACTGCCTGGTGATACGAGGATGGGCATGCTCTTCCCG‐3′
Cy5‐S1: 5′‐Cy5‐ATTTATCACCCGCCATAGTAGACGTATCACCAGGCAGTTGAGACGAACATTCCTAAGTCTGAA‐3′

### 2.2 Preparation and characterization of tFNAs complexes

We added the presynthesized nucleic acid single strand to a buffer solution (10 mM Tris and 5 mM MgCl_2_) at a final concentration of 1 μM per single chain, vortexed it, and placed it in a PCR machine. The mixture was heated at 95°C for 20 min and then cooled to 4°C for 20 min. The successful synthesis of tFNAs complexes was validated by non‐denaturing polyacrylamide gel electrophoresis (SDS‐PAGE) and dynamic light scattering techniques (DLS).

### 2.3 Culture of ADSCs

All of the operations involving animals were in compliance with the requirements of the Ethics Committee. We extracted adipose stem cells from the 5‐day‐old SD rat inguinal adipose tissue. Specifically, we crushed the obtained adipose tissue and digested it with type I collagenase for 45 min at 37°C. Subsequently, the mixture was centrifuged at 200*g* for 6 min, and then the supernatant was discarded. The cells were then resuspended by a conventional growth medium consisting of α‐MEM (Gibco, CA), 100 U/ml penicillin/streptomycin (Gibco, CA), and 10% fetal bovine serum. Finally, the cells were inoculated in a culture flask. We changed the culture medium every 2 days. Cells in the third passage were used for subsequent experiments.

### 2.4 Detection of the ability of different materials to enter cells

Compared with single‐stranded DNA, which requires the assistance of another vector to enter the cells, the tFNAs nanomaterial can enter the cells due to its three‐dimensional structure, thereby exerting corresponding biological effects. To detect whether the synthetic novel tFNAs complex can be successfully inserted into the cells, we ligated the CY‐5 fluorescent molecule on the single strand of the nucleic acid. Then, we detected the ability of different materials to enter cells by immunofluorescence and flow cytometry. The obtained ADSCs were seeded on a six‐well plate. Then, after 24 h of culture, single nucleic acid single strands, tFNAs, and tFNAs–miR‐26a complex were separately added into the cells for 12 h. Then, we carried out the corresponding test.

### 2.5 Osteogenic induction of ADSCs

An OriCell Rat ADSCs Osteogenic Differentiation Kit (Cyagen Biosciences Inc., Goleta, CA) was used to detect the effect of tFNAs–miR‐26a on osteogenic differentiation ability of ADSCs. We inoculated ADSCs in a six‐well plate (1 × 10^5^ cells per well, 2 ml medium per well), and cultured them for 3 days. When the density reached 85%–90%, ADSCs were starved with α‐MEM containing 1% FBS for 2 h and then treated with 250 nm tFNAs–miR‐26a for 48 h. Afterwards, the cells were gently rinsed with phosphate buffer solution (PBS), and the medium was substituted with OriCell Rat ADSCs complete medium for osteogenic induction.

### 2.6 Real‐time polymerase chain reaction

In order to detect the effect of tFNAs–miR‐26a on the expression of osteogenic‐specific genes, we used real‐time polymerase chain reaction (RT‐PCR) technology to detect the expression of osteopontin (OPN), alkaline phosphatase (ALP), and runt‐related transcription factor 2 (RUNX2). As shown in Table 2, we also detected the expression of genes related to the Wnt signaling pathway, such as β‐catenin, glycogen synthase kinase (GSK) and lymphatic enhancement factor‐1 (Lef‐1). After treatment with tFNAs–miR‐26a for 1 day, TRIzol reagent (Thermo Fisher Scientific, MA) was employed to extract gene samples. Subsequently, we used a cDNA synthesis kit (Mbi, Glen Burnie, MD) to purify and obtain the cDNA, and then we employed SYBR Green I PCR master mix and the Bio‐Rad real‐time PCR system (Bio‐Rad, Hercules, CA) to perform qPCR.

**TABLE 2:** Primer sequences used to detect the relevant genes by qPCR

Genes	Lengths (bp)	Primer (5′–3′)	Sequence
*GAPDH*	233	Forward Reverse	ACAGCAACAGGGTGGTGGAC TTTGAGGGTGCAGCGAACTT
*GSK‐3β*	266	Forward Reverse	GCAGATCATGCGTAAGCTGGAC GGTACACTGTCTCGGGCACATA
*β‐Catenin*	281	Forward Reverse	AAGTTCTTGGCTATTACGACA ACAGCACCTTCAGCACTCT
*Lef‐1*	120	Forward Reverse	ACAGATCACCCCACCTCTTG TGATGGGAAAACCTGGACAT
*ALP*	106	Forward Reverse	ATCTTTGGTCTGGCTCCCATG TTTCCCGTTCACCGTCCAC
*OPN*	160	Forward Reverse	CACTCCAATCGTCCCTACA CTTAGACTCACCGCTCTTCAT
*Runx 2*	137	Forward Reverse	AGGGACTATGGCGTCAAACA GGCTCACGTCGCTCATCTT

### 2.7 Western blot

In order to explore the effect of tFNAs–miR‐26a complex on osteogenic differentiation of ADSCs, the protein expression levels of OPN and RUNX2 were detected by western blot. In addition, to examine the relevant mechanism, the expression levels of the Wnt signaling pathway‐related proteins were also examined. A cell protein extraction reagent (KeyGen Biotech, Nanjing, China) was used to harvest total proteins. All of the collected samples were added to 5× loading buffer (Beyotime, Shanghai, China), mixed well, placed in boiling water for 5 min to allow albumin denaturation, and then finally stored at −20°C. Anti‐OPN (Abcam, Cambridge, UK), anti‐Runx2 (Abcam, Cambridge, UK), anti‐Lef‐1 (Abcam, Cambridge, UK), anti‐GSK (Abcam, Cambridge, UK), anti‐cyclin D (Abcam, Cambridge, UK), and anti‐β‐catenin (Abcam, Cambridge, UK) were incubated with the protein samples overnight. The second day, samples were incubated with relevant secondary antibody (Beyotime, Shanghai, China). Finally, an ECL reagent (Millipore, MA) was used to visualize the protein bands.

### 2.8 Cellular immunofluorescence

To further verify the ability of tFNAs–miR‐26a to promote the expression of the osteogenesis‐related proteins and the key proteins of the Wnt signaling pathway, we used immunofluorescence technology to detect the expression levels of the related proteins. The cells were seeded and treated in groups in line with the above method. At the corresponding time point, the supernatant was discarded; the plate was washed with PBS, fixed with paraformaldehyde for 15 min, treated with 0.5% TritonX‐100 for 20 min, and blocked with 0.5% goat serum at room temperature for 20 min; and then the plate was incubated with the relevant primary antibodies (anti‐Runx2, anti‐OPN, anti‐GSK, and anti‐β‐catenin) overnight at 4°C. The next day, the samples were incubated with secondary antibodies at 37°C for 1 h. We stained nuclei and cytoplasm with DAPI and phalloidin, respectively, and the samples were finally mounted with 10% glycerol. Finally, a confocal laser microscope (LSM700; Zeiss, Oberkochen, Germany) were used to capture images.

### 2.9 Statistical analysis

Student's *t* test or one‐way ANOVA in GraphPad Prism version 8.0.2 (GraphPad Soft‐ware Inc., San Diego, CA) were used for statistical analysis.

## 3 RESULTS

### 3.1 Preparation and characterization of materials

**FIGURE 1:**
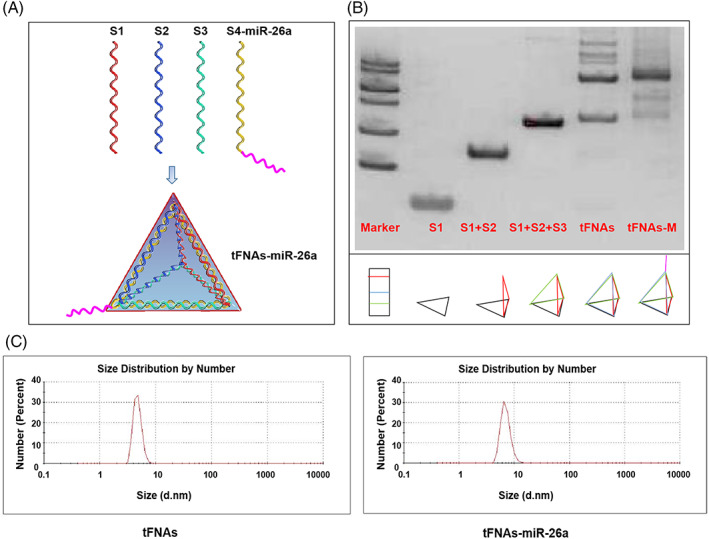
Successful synthesis of tFNAs–miR‐26a. (A) Sketch map of tFNAs–miR‐26a. (B) Confirmation of the successful synthesis of tFNAs–miR‐26a by native SDS‐PAGE (M: marker, S: single‐stranded DNA). (C) Confirmation of the successfully assembled tFNAs–miR‐26a by dynamic light scattering.

Through relevant literature searches, miR‐26a, which can significantly promote osteogenic differentiation of ADSCs, was screened out. When designing the single‐stranded S4 chain of tFNAs, four thy mines were added to the end, and then the base sequence of miR‐26a was connected. The synthesis conditions of tFNAs that had been explored in previous experiments were used to prepare new tFNAs carrying miR‐26a. As shown in Figure 1A, the tFNAs–miR‐26a complex was formed by the self‐assembly of four single strands (S1, S2, S3, and S4‐miR‐26a) through the principle of complementary base pairing, and each single strand was connected end‐to‐end to form a face of the tetrahedral structure. Subsequently, we used native SDS‐PAGE to verify the successful synthesis of the complex (as shown in Figure 1B). Finally, DLS was used to detect the particle size of the material. The detection results showed that the particle size of tFNAs was about 7 nm, and the particle size of the tFNAs–miR‐26a complex was about 8 nm, which is consistent with the results reported in the literature (Figure 1C).

### 3.2 Cellular membrane crossing of the tFNAs–miR‐26a complex

**FIGURE 2:**
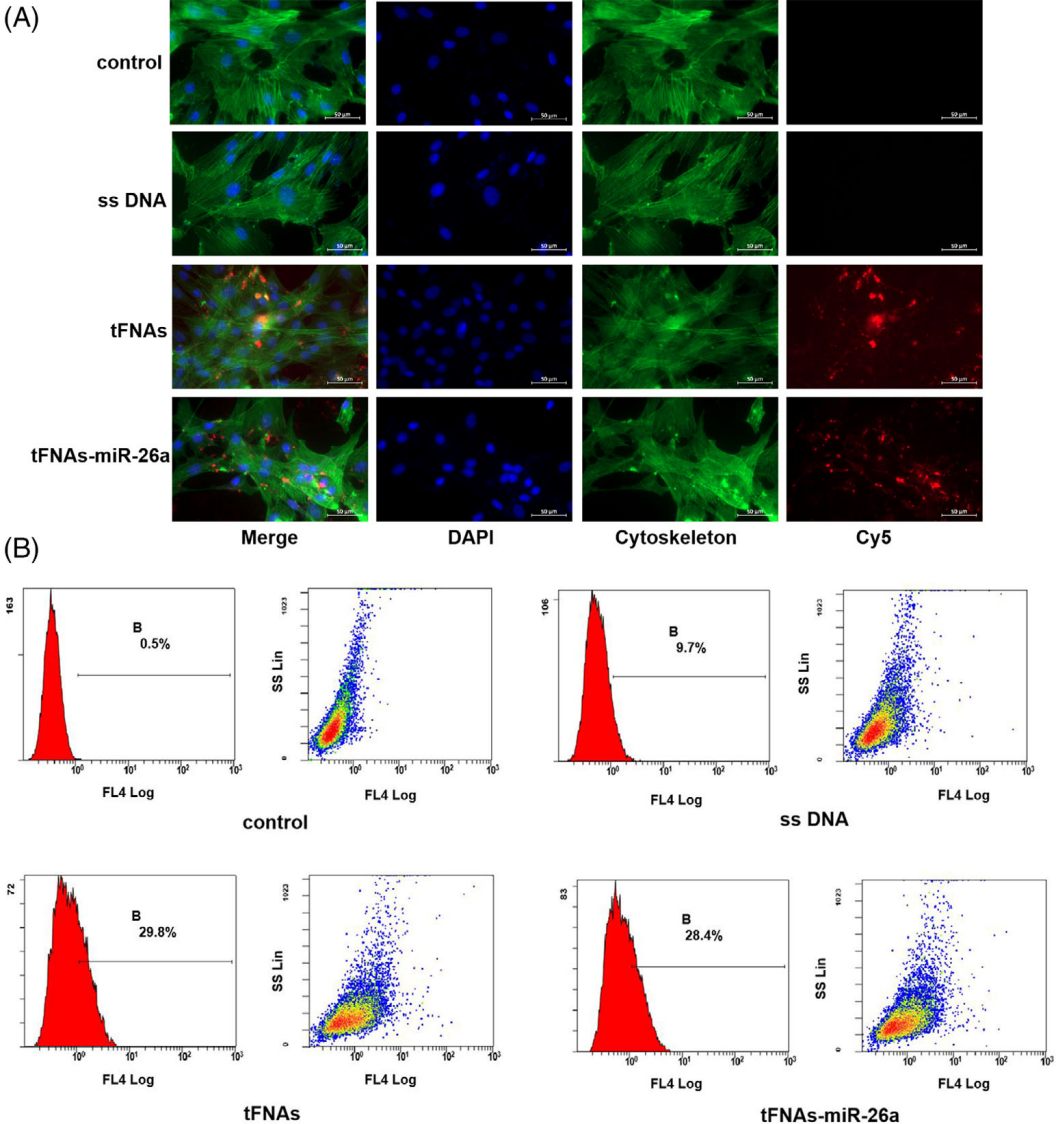
Cellular uptake of tFNAs–miR‐26a. (A) Cellular uptake of Cy5‐tFNAs–miR‐26a, Cy5‐tFNAs, and Cy5‐single‐stranded DNA in ADSCs (Cytoplasm: green; Nucleus: blue; Cy5: red). Scale bars are 50 μm. (B) Cellular uptake of Cy5‐tFNAs–miR‐26a, Cy5‐tFNAs, and Cy5‐single‐stranded DNA by flow cytometry.

To further confirm that the tFNAs–miR‐26a complex, rather than the DNA single strand, plays the corresponding biological role in ADSCs, we used flow cytometry and immunofluorescence techniques to evaluate the cell entrapment ability of the material. The detection results of flow cytometry showed that tFNAs and tFNAs–miR‐26a complexes had better ability to enter cells than DNA single strands, which had difficulty entering ADSCs (Figure 2B). The results of immunofluorescence detection (Figure 2A) further demonstrated the infiltration of the material, which was consistent with the results of flow cytometry detection. The above results suggest that the tFNAs–miR‐26a complex has a good ability to enter ADSCs, which lays a solid foundation for the subsequent experiments to detect the role of the tFNAs–miR‐26a complex in inducing osteogenic differentiation of ADSCs.

### 3.3 Promotion of osteogenic differentiation

**FIGURE 3:**
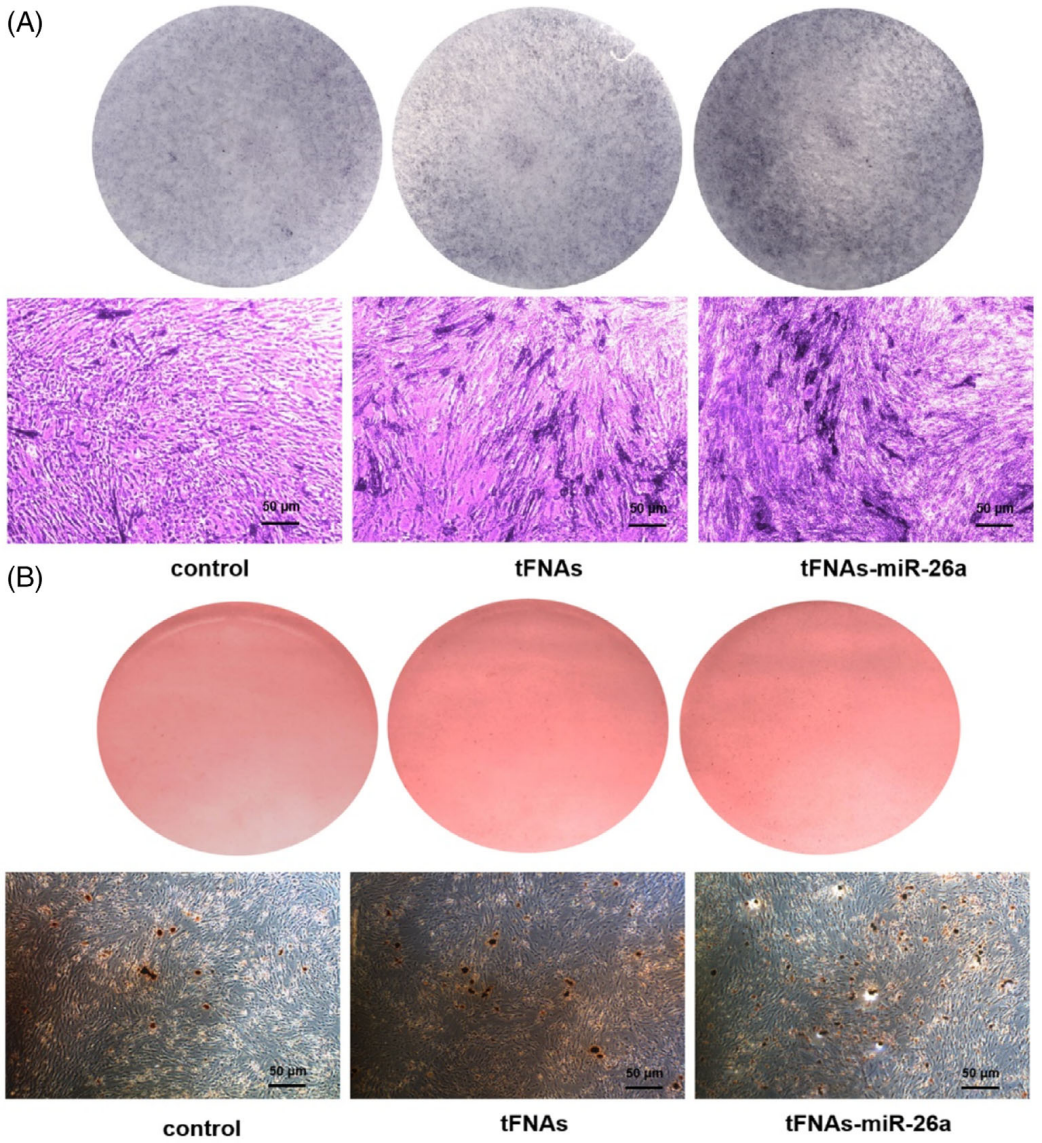
Enhancement of ALP activity and calcium nodules formation after exposure to tFNAs–miR‐26a. (A) Osteogenic differentiation was detected by ALP staining (top) at day 7 and NBT‐formation in ALP‐stained cells after 7 days of osteogenic differentiation (bottom). (B) Osteogenic differentiation was detected by Alizarin Red staining (top) at day 15 and calcium nodules in Alizarin Red‐stained cells after osteogenic induction for 15 days (bottom).

The cells were grouped to Ctrl, tFNAs, and tFNAs–miR‐26a for subsequent experiments. To examine the effect of the complex on the osteogenic differentiation ability of ADSCs, we measured the early osteogenic differentiation marker ALP activity using the BCIP/NBT alkaline phosphatase chromogenic kit 7 days after the osteogenic differentiation treatment. Compared with the nontreated group and the tFNAs group, the ALP activity was significantly enhanced after treatment with the tFNAs–miR‐26a complex, and more nitro blue tetrazolium (NBT) formation was found (Figure 3A). To further explore whether tFNAs–miR‐26a can promote the differentiation ability of ADSCs, we applied Alizarin Red to stain calcium nodules formed after 15 days of osteogenic induction culture (Figure 3B). Calcium nodules are considered the late markers of osteogenic differentiation of adipose‐derived mesenchymal stem cells. In this part of the experiment, we found that more calcium nodules were formed after treatment with tFNAs–miR‐26a, which further confirmed that tFNAs–miR‐26a had a certain effect on the osteogenic differentiation ability of ADSCs.

### 3.4 Promotion of the expression of osteogenic differentiation‐specific genes and proteins

**FIGURE 4:**
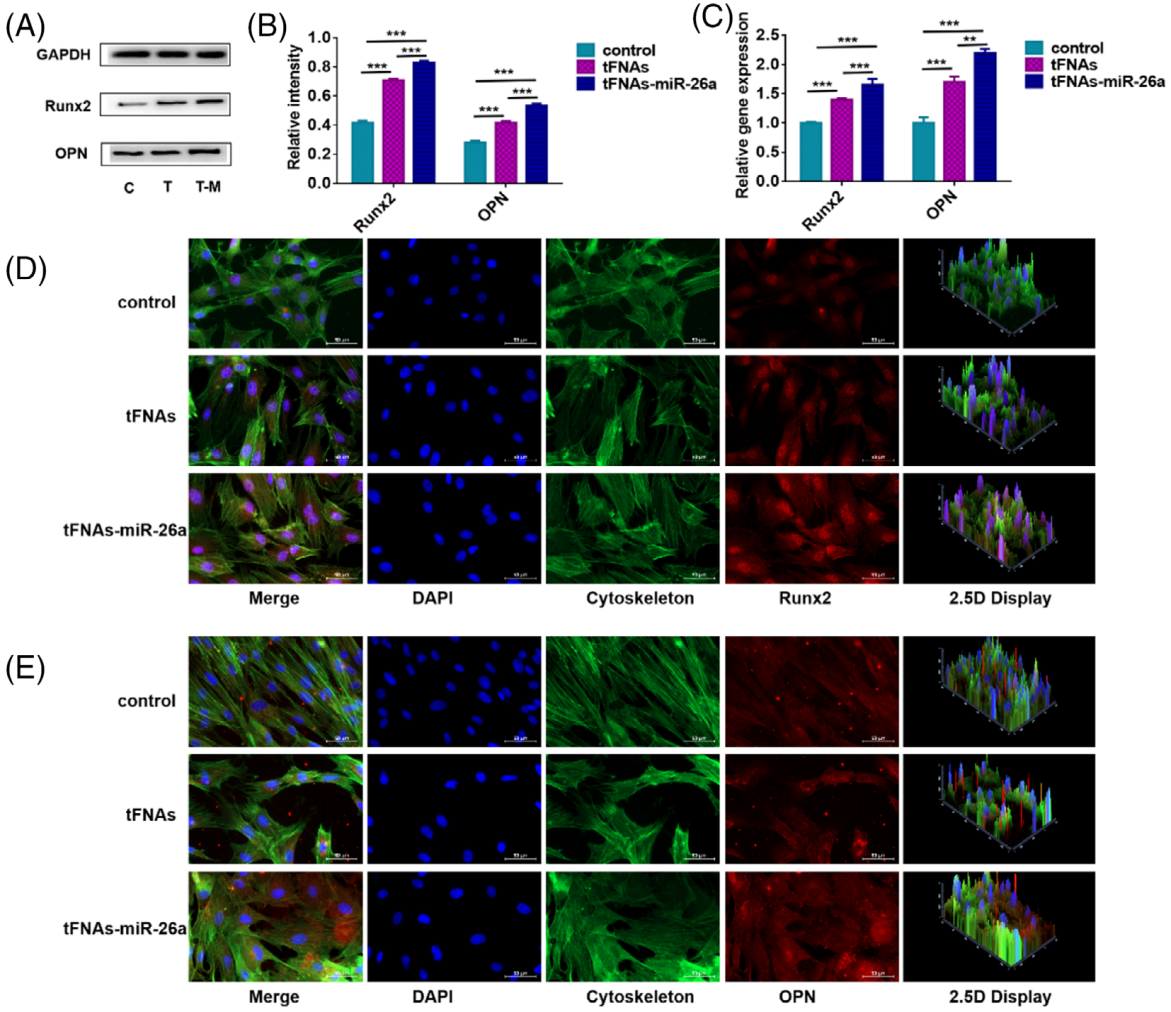
Detection of osteogenic differentiation‐specific proteins and genes. (A) Western blot analysis of protein expression levels upon exposure to tFNAs–miR‐26a (250 nM) for 24 h. (B) Quantification of protein expression levels upon exposure to tFNAs–miR‐26a (250 nM) for 24 h. Data are presented as mean ± SD (*n* = 4). Student's *t* test: **p < 0.01, ***p < 0.001. (C) Quantification of gene expression levels upon exposure to tFNAs–miR‐26a (250 nM) for 24 h. Data are presented as mean ± SD (*n* = 4). Student's *t* test: **p < 0.01, ***p < 0.001. (D) Photomicrographs showing treated ADSCs (Cytoplasm: green, Nucleus: blue, Runx2: red). Scale bars are 50 μm. (E) Photomicrographs showing treated ADSCs (Cytoplasm: green, Nucleus: blue, OPN: red). Scale bars are 50 μm.

The previous experimental results showed that tFNAs–miR‐26a had a certain effect on the expression of early and late osteogenic differentiation markers of ADSCs. To further confirm the effect of tFNAs–miR‐26a on the osteogenic differentiation ability of ADSCs, we detected the expression levels of osteogenic differentiation‐specific genes and proteins (ALP, Runx2, and OPN). We examined the expression of these osteogenic‐specific genes in different treatment groups after 7 days of osteogenic differentiation. As shown in Figure 4C, the expression of the specific genes related to osteogenic differentiation of these ADSCs was significantly upregulated after treatment with the tFNAs–miR‐26a complex. Specifically, the expression levels of OPN were 2.21 times higher than those in the control group. In addition, the expression of Runx2 was increased to 1.63‐fold compared with the control group (Figure 4C).

In addition to detecting osteogenic differentiation‐related genes of ADSCs, we also detected the expression levels of osteogenic differentiation‐related proteins by western blot and immunofluorescence techniques. After treatment with the tFNAs–miR‐26a complex, we performed immunofluorescence staining on the samples. By confocal laser microscopy, we detected that OPN and Runx2 showed stronger fluorescent signals in the tFNAs–miR‐26a‐treated group than in the control group (Figure 4D,E). In addition, by western blot, we detected the expression changes of osteogenic‐specific proteins after tFNAs–miR‐26a treatment. As shown in Figure 4A, the protein expression levels of OPN and Runx2 were significantly enhanced after the tFNAs–miR‐26a complex treatment. Statistical analysis further confirmed this conclusion (1.85‐fold increase in OPN and 2.05‐fold increase in Runx2, Figure 4B). Taken together, these results suggest that the tFNAs–miR‐26a complex can promote osteogenic differentiation of ADSCs and significantly upregulate the expression of osteogenic differentiation‐specific proteins and genes.

### 3.5 tFNAs–miR‐26a promotes osteogenic differentiation of ADSCs by downregulating the expression of GSK‐3β

**FIGURE 5:**
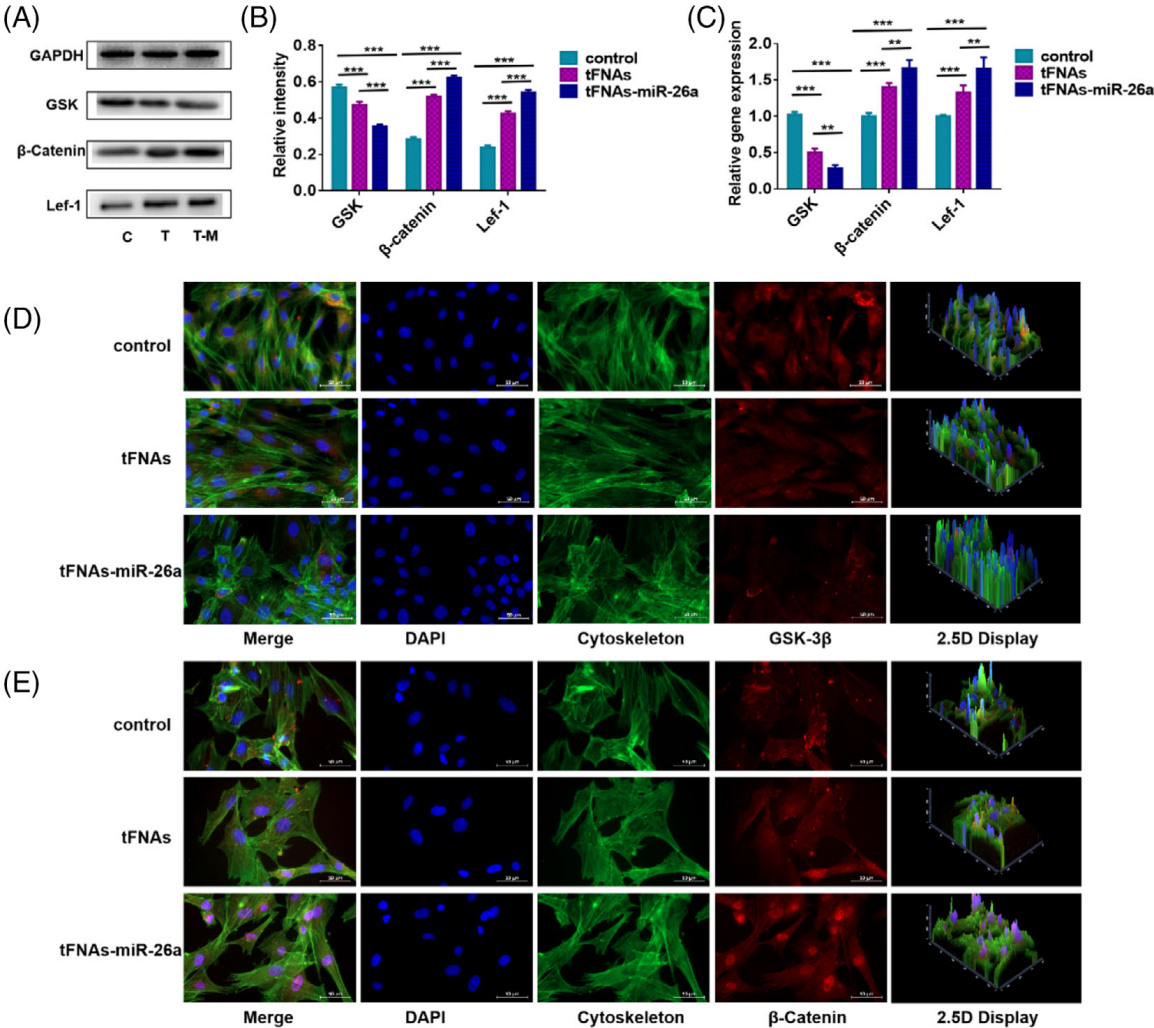
Detection of canonical Wnt/β‐catenin signaling pathway related proteins and genes. (A) Western blot analysis of protein expression levels upon exposure to tFNAs–miR‐26a (250 nM) for 24 h. (B) Quantification of protein expression levels upon exposure to tFNAs–miR‐26a (250 nM) for 24 h. Data are presented as mean ± SD (n = 4). Student's *t* test: **p < 0.01, ***p < 0.001. (C) Quantification of gene expression levels upon exposure to tFNAs–miR‐26a (250 nM) for 24 h. Data are presented as mean ± SD (n = 4). Student's *t* test: **p < 0.01, ***p < 0.001. (D) Photomicrographs showing treated ADSCs (cytoplasm: green, nucleus: blue, GSK‐3β: red). Scale bars are 50 μm. (E) Photomicrographs showing treated ADSCs (Cytoplasm: green, Nucleus: blue, β‐Catenin: red). Scale bars are 50 μm.

To further study the regulatory mechanism of tFNAs–miR‐26a in promoting osteogenic differentiation of mesenchymal stem cells, three miRNA target gene prediction databases (PicTar (http://pictar.mdc-berlin.de/), TargetScan (http://www.targetscan.org/mmu_50/), and TargetRank (http://genes.mit.edu/targetrank/)) were used to predict the target genes of miR‐26a. Among the predicted target genes, GSK‐3β was considered to be closely related to osteogenic differentiation. Subsequently, the gene and protein expression of GSK‐3β was detected after tFNAs–miR‐26a treatment of ADSCs. The results showed that compared with the blank control group and the unintentional sequence group without material treatment, the expression of GSK‐3β was significantly downregulated in the process of regulating osteogenic differentiation of ADSCs with tFNAs–miR‐26a. It has been reported in the literature that GSK‐3β is a key enzyme of the Wnt pathway, and the canonical Wnt/β‐catenin signaling pathway regulates osteogenic differentiation, bone matrix formation, and mineralization of mesenchymal stem cells by regulating downstream osteogenesis‐related transcription factors. When the expression of GSK‐3β decreases, a large amount of β‐catenin accumulates in the cytoplasm, and the excess β‐catenin is translocated to the nucleus to interact with Lef‐1 and subsequently regulate the expression of Runx2, which can promote mesenchymal stem cells osteogenic differentiation. Subsequently, we used RT‐PCR, western blot, and immunofluorescence to detect the expression levels of the Wnt signaling pathway‐related genes and proteins. As shown by PCR (Figure 5C), tFNAs–miR‐26a treatment led to a significant increase in the expression of β‐catenin and Lef‐1 (1.65‐fold increase for β‐catenin and 1.71‐fold increase for Lef‐1). Then, we examined the expression levels of the related proteins. As shown in Figure 5A, the protein expression levels of β‐catenin and Lef‐1 were significantly enhanced after adding tFNAs–miR‐26a. After tFNAs–miR‐26a treatment, compared with the blank control group, the protein expression level of β‐catenin increased by 2.52 times, that of Lef‐1 increased by 2.71 times, and that of GSK decreased by 1.81 times (Figure 5B). To further confirm the role of the Wnt/β‐catenin signaling pathway, we visualized the changes in β‐catenin protein expression by immunofluorescence staining. We showed that after adding tFNAs‐miR‐26a, β‐catenin protein in ADSCs showed a stronger fluorescent signal (Figure 5D). In conclusion, tFNAs‐miR‐26a may play an important role in osteogenic differentiation of ADSCs by downregulating the expression of GSK‐3β and activating the canonical Wnt/β‐catenin signaling pathway.

## 4 DISCUSSION

Maxillofacial trauma, tumors, and congenital deformities are common causes of craniofacial bone tissue defects. Craniomaxillofacial bones are crucial to maintaining facial beauty, chewing, swallowing, language and other functions, and craniomaxillofacial bone defects can easily lead to serious psychological problems, causing great physical and mental pain to patients and a great burden to the patient's family.^31^ The biggest obstacle to the clinical application of the existing craniofacial bone tissue defect repair materials is that the tissue materials lack good osteoinductive and osteogenic properties, so they cannot obtain sufficient amount of mature new bone in a short period of time.^32^, ^33^ With the rapid development of molecular biology, regenerative biology, and genetic engineering technology, it is of great significance to combine stem cells, biomaterials, and growth factors to regulate cell regeneration, thereby promoting the repair of defective tissues.^34^ Previous studies have reported that some miRNAs (miR‐133, miR‐135, miR‐138, miR‐637, and miR‐26a) are abnormally expressed during osteogenic differentiation of mesenchymal stem cells, indicating that some miRNAs are involved in the repair of bone tissue defects.^35^, ^36^ However, the disadvantages of miRNAs, such as poor stability, inability to penetrate cell membranes, and low specificity, severely restrict their applications.^9^ At present, the commonly used miRNA delivery vehicles include viral transfection and liposome delivery, but the biological safety of viral transfection is low, and the use of liposomes as miRNA delivery vehicles confers certain cytotoxicity.^37^ Therefore, finding an ideal carrier to safely and efficiently transfect exogenous miRNAs that can promote osteogenic differentiation into ADSCs, and conduct in‐depth research on the specific regulatory mechanism has become an important breakthrough in the regeneration and treatment of bone tissue defects. In this study, when we designed the single‐stranded S4 chain of tFNAs, we added five thymines to its end, and then linked the base sequence of miR‐26a. Subsequently, a novel tFNAs–miR‐26a complex was prepared by applying the synthetic conditions of tFNAs that had been explored in previous experiments.^24^ After tFNAs carry miR‐26a into cells, the five thymines at the junction of the complex are digested by intracellular nucleases, and tFNAs and miRNAs are released intracellularly and exert their biological functions. The subsequent experimental results revealed that tFNAs can carry miR‐26a into ADSCs, and that tFNAs–miR‐26a can effectively increase the expression of the key growth factors related to osteogenic differentiation (ALP, OPN, and Runx2) at both gene and protein levels, thereby improving the activity of alkaline phosphatase and mineralization ability of ADSCs, and further promoting ADSCs' osteogenic differentiation. MiR‐26a can play an important regulatory role in the osteogenic differentiation of ADSCs, and can effectively promote the expression of key growth factors ALP, OPN and Runx2 related to osteogenic differentiation at the gene and protein levels. In the in vivo environment, the ideal effect of bone defect repair can be achieved by overexpressing miR‐26a in the defect area and its surrounding cells.^8^, ^9^ What are the target genes and regulatory networks of tFNAs–miR‐26a‐induced osteogenic differentiation of ADSCs? To further verify the mechanism of tFNAs–miR‐26a‐induced osteogenic differentiation of ADSCs, we examined the canonical Wnt signaling pathway, which is considered to be a key regulator of mesenchymal stem cells. The canonical Wnt/β‐catenin signaling pathway plays an important role in the process of osteogenic differentiation.^38^ Our results showed that after tFNAs–miR‐26a treatment, the osteogenic differentiation ability of ADSCs was enhanced, which was accompanied by the downregulation of GSK‐3β and the increased expression of the genes and proteins related to the canonical Wnt/β‐catenin signaling pathway. Alkaline phosphatase staining and calcium deposition experiments showed that tFNAs‐miR‐26a could play a significant role in promoting bone formation by enhancing alkaline phosphatase activity and stimulating matrix mineralization.

In summary, our experimental results confirmed that tFNAs can serve as excellent delivery vehicles for miR‐26a, to carry miR‐26a into ADSCs and improve the targeting of miRNA effects; moreover, tFNAs can be used in the process of bone tissue defect repair and treatment, combined with miR‐26a to promote osteogenic differentiation of ADSCs, thereby promoting the formation of sufficiently mature new bone. The tFNAs–miR‐26a complex can activate the canonical Wnt/β‐catenin signaling pathway by downregulating the expression of GSK‐3β, and it can promote the expression of the osteogenic differentiation–specific genes ALP, OPN, and Runx2 in ADSCs. This study promotes the formation of sufficiently mature new bone from the perspective of constructing a novel microRNA delivery system that promotes osteogenic differentiation of ADSCs, and it provides new ideas for bone defect repair.

## AUTHOR CONTRIBUTIONS

All authors contributed to the study concept and design. Xiaoru Shao and Zhong Hu carried out the in vitro experiments on ADSCs. Wenjuan Ma and Yuxi Zhan collected the data. Xiaoru Shao performed the analysis and drafted the manuscript. Li Quan participated in the detection and data analysis of our related experiments. Yunfeng Lin initiated the research, designed research studies and analyzed data. All authors have reviewed and approved the manuscript.

## CONFLICT OF INTEREST

The authors declare no competing interests.

## Data Availability

The data that support the findings of this study are available from the corresponding author upon reasonable request.

## References

[1] Cates K , McCoy MJ , Kwon JS , et al. Deconstructing stepwise fate conversion of human fibroblasts to neurons by microRNAs. Cell Stem Cell. 2021;28:127‐140.e9. 32961143 10.1016/j.stem.2020.08.015 PMC7796891

[2] Xu XH , Yuan TJ , Dad HA , et al. Plant exosomes as novel nanoplatforms for microRNA transfer stimulate neural differentiation of stem cells in vitro and in vivo. Nano Lett. 2021;21:8151‐8159. 34586821 10.1021/acs.nanolett.1c02530

[3] Wosczyna MN , Perez Carbajal EE , Wagner MW , et al. Targeting microRNA‐mediated gene repression limits adipogenic conversion of skeletal muscle mesenchymal stromal cells. Cell Stem Cell. 2021;28:1323‐1334.e8. 33945794 10.1016/j.stem.2021.04.008 PMC8254802

[4] Zhang D , Ni N , Wang Y , et al. CircRNA‐vgll3 promotes osteogenic differentiation of adipose‐derived mesenchymal stem cells via modulating miRNA‐dependent integrin alpha5 expression. Cell Death Differ. 2021;28:283‐302. 32814879 10.1038/s41418-020-0600-6 PMC7853044

[5] Li B . MicroRNA regulation in osteogenic and adipogenic differentiation of bone mesenchymal stem cells and its application in bone regeneration. Curr Stem Cell Res Ther. 2018;13:26‐30. 28578644 10.2174/1574888X12666170605112727

[6] Duan DY , Tang J , Tian HT , Shi YY , Jia J . Adipocyte‐secreted microvesicle‐derived miR‐148a regulates adipogenic and osteogenic differentiation by targeting Wnt5a/Ror2 pathway. Life Sci. 2021;278:119548. 33930365 10.1016/j.lfs.2021.119548

[7] Li Y , Fan L , Hu J , et al. MiR‐26a rescues bone regeneration deficiency of mesenchymal stem cells derived from osteoporotic mice. Mol Ther. 2015;23:1349‐1357. 26050992 10.1038/mt.2015.101 PMC4817873

[8] Li S , Hu C , Li J , et al. Effect of miR‐26a‐5p on the Wnt/Ca(^2+^) pathway and osteogenic differentiation of mouse adipose‐derived mesenchymal stem cells. Calcif Tissue Int. 2016;99:174‐186. 27040676 10.1007/s00223-016-0137-3

[9] Zhao Z , Dai XS , Wang ZY , Bao ZQ , Guan JZ . MicroRNA‐26a reduces synovial inflammation and cartilage injury in osteoarthritis of knee joints through impairing the NF‐kappaB signaling pathway. Biosci Rep. 2019;39:BSR20182025. 10.1042/BSR20182025 PMC6454017 30872407

[10] Basu S , Pacelli S , Feng Y , Lu Q , Wang J , Paul A . Harnessing the noncovalent interactions of DNA backbone with 2D silicate nanodisks to fabricate injectable therapeutic hydrogels. ACS Nano. 2018;12:9866‐9880. 30189128 10.1021/acsnano.8b02434 PMC6563937

[11] Tan B , Tang Q , Zhong Y , et al. Biomaterial‐based strategies for maxillofacial tumour therapy and bone defect regeneration. Int J Oral Sci. 2021;13:9. 33727527 10.1038/s41368-021-00113-9 PMC7966790

[12] El‐Rashidy AA , Roether JA , Harhaus L , Kneser U , Boccaccini AR . Regenerating bone with bioactive glass scaffolds: a review of in vivo studies in bone defect models. Acta Biomater. 2017;62:1‐28. 28844964 10.1016/j.actbio.2017.08.030

[13] Daly AC , Freeman FE , Gonzalez‐Fernandez T , Critchley SE , Nulty J , Kelly DJ . 3D bioprinting for cartilage and osteochondral tissue engineering. Adv Healthc Mater. 2017;6:1700298. 10.1002/adhm.201700298 28804984

[14] Dalisson B , Charbonnier B , Aoude A , et al. Skeletal regeneration for segmental bone loss: vascularised grafts, analogues and surrogates. Acta Biomater. 2021;136:37‐55. 34626818 10.1016/j.actbio.2021.09.053

[15] Sirong S , Yang C , Taoran T , et al. Effects of tetrahedral framework nucleic acid/wogonin complexes on osteoarthritis. Bone Res. 2020;8:6. 32047705 10.1038/s41413-019-0077-4 PMC7010777

[16] Galbraith T , Clafshenkel WP , Kawecki F , et al. A cell‐based self‐assembly approach for the production of human osseous tissues from adipose‐derived stromal/stem cells. Adv Healthc Mater. 2017;6:1600889. 10.1002/adhm.201600889 28004524

[17] Ko E , Lee JS , Kim H , et al. Electrospun silk fibroin nanofibrous scaffolds with two‐stage hydroxyapatite functionalization for enhancing the osteogenic differentiation of human adipose‐derived mesenchymal stem cells. ACS Appl Mater Interfaces. 2018;10:7614‐7625. 28475306 10.1021/acsami.7b03328

[18] Xia L , Lin K , Jiang X , et al. Effect of nano‐structured bioceramic surface on osteogenic differentiation of adipose derived stem cells. Biomaterials. 2014;35:8514‐8527. 25002263 10.1016/j.biomaterials.2014.06.028

[19] Taha MF , Hedayati V . Isolation, identification and multipotential differentiation of mouse adipose tissue‐derived stem cells. Tissue Cell. 2010;42:211‐216. 20483444 10.1016/j.tice.2010.04.003

[20] Zhang T , Tian T , Zhou R , et al. Design, fabrication and applications of tetrahedral DNA nanostructure‐based multifunctional complexes in drug delivery and biomedical treatment. Nat Protoc. 2020;15:2728‐2757. 32669637 10.1038/s41596-020-0355-z

[21] Wang Y , Li Y , Gao S , Yu X , Chen Y , Lin Y . Tetrahedral framework nucleic acids can alleviate taurocholate‐induced severe acute pancreatitis and its subsequent multiorgan injury in mice. Nano Lett. 2022;22:1759‐1768. 35138113 10.1021/acs.nanolett.1c05003

[22] Zhu J , Yang Y , Ma W , et al. Antiepilepticus effects of tetrahedral framework nucleic acid via inhibition of gliosis‐induced downregulation of glutamine synthetase and increased AMPAR internalization in the postsynaptic membrane. Nano Lett. 2022;22:2381‐2390. 35266400 10.1021/acs.nanolett.2c00025

[23] Ma W , Yang Y , Zhu J , et al. Biomimetic nanoerythrosome‐coated aptamer‐DNA tetrahedron/maytansine conjugates: pH‐responsive and targeted cytotoxicity for HER2‐positive breast cancer. Adv Mater. 2022;e2109609. 35064993 10.1002/adma.202109609

[24] Zhang T , Tian T , Lin Y . Functionalizing framework nucleic‐acid‐based nanostructures for biomedical application. Adv Mater. 2021;e2107820. 34787933 10.1002/adma.202107820

[25] Liedl T , Hogberg B , Tytell J , Ingber DE , Shih WM . Self‐assembly of three‐dimensional prestressed tensegrity structures from DNA. Nat Nanotechnol. 2010;5:520‐524. 20562873 10.1038/nnano.2010.107 PMC2898913

[26] Zhou M , Zhang T , Zhang B , et al. A DNA nanostructure‐based neuroprotectant against neuronal apoptosis via inhibiting toll‐like receptor 2 signaling pathway in acute ischemic stroke. ACS Nano. 2021;16:1456‐1470. 10.1021/acsnano.1c09626 34967217

[27] Zhang M , Zhang X , Tian T , et al. Anti‐inflammatory activity of curcumin‐loaded tetrahedral framework nucleic acids on acute gouty arthritis. Bioact Mater. 2022;8:368‐380. 34541407 10.1016/j.bioactmat.2021.06.003 PMC8429917

[28] Zhou M , Gao S , Zhang X , et al. The protective effect of tetrahedral framework nucleic acids on periodontium under inflammatory conditions. Bioact Mater. 2021;6:1676‐1688. 33313447 10.1016/j.bioactmat.2020.11.018 PMC7708773

[29] Liu Y , Sun Y , Li S , et al. Tetrahedral framework nucleic acids deliver antimicrobial peptides with improved effects and less susceptibility to bacterial degradation. Nano Lett. 2020;20:3602‐3610. 32272018 10.1021/acs.nanolett.0c00529

[30] Qin X , Xiao L , Li N , et al. Tetrahedral framework nucleic acids‐based delivery of microRNA‐155 inhibits choroidal neovascularization by regulating the polarization of macrophages. Bioact Mater. 2022;14:134‐144. 35310341 10.1016/j.bioactmat.2021.11.031 PMC8892086

[31] Xue Y , Zhu Z , Zhang X , et al. Accelerated bone regeneration by MOF modified multifunctional membranes through enhancement of osteogenic and angiogenic performance. Adv Healthc Mater. 2021;10:e2001369. 33448103 10.1002/adhm.202001369

[32] Wang CW , Yu SH , Fretwurst T , et al. Maresin 1 promotes wound healing and socket bone regeneration for alveolar ridge preservation. J Dent Res. 2020;99:930‐937. 32384864 10.1177/0022034520917903 PMC7338694

[33] Dubey N , Ferreira JA , Daghrery A , et al. Highly tunable bioactive fiber‐reinforced hydrogel for guided bone regeneration. Acta Biomater. 2020;113:164‐176. 32540497 10.1016/j.actbio.2020.06.011 PMC7482137

[34] Salamanca E , Hsu CC , Yao WL , et al. Porcine collagen‐bone composite induced osteoblast differentiation and bone regeneration in vitro and in vivo. Polymers (Basel). 2020;12:93. 10.3390/polym12010093 PMC7023633 31947902

[35] Hodges WM , O'Brien F , Fulzele S , Hamrick MW . Function of microRNAs in the osteogenic differentiation and therapeutic application of adipose‐derived stem cells (ASCs). Int J Mol Sci. 2017;18:2597. 10.3390/ijms18122597 PMC5751200 29207475

[36] Yang S , Guo S , Tong S , Sun X . Promoting osteogenic differentiation of human adipose‐derived stem cells by altering the expression of exosomal miRNA. Stem Cells Int. 2019;2019:1351860‐1351815. 31354836 10.1155/2019/1351860 PMC6636464

[37] Maestro S , Weber ND , Zabaleta N , Aldabe R , Gonzalez‐Aseguinolaza G . Novel vectors and approaches for gene therapy in liver diseases. JHEP Rep. 2021;3:100300. 34159305 10.1016/j.jhepr.2021.100300 PMC8203845

[38] Abuna RPF , Oliveira FS , Adolpho LF , Fernandes RR , Rosa AL , Beloti MM . Frizzled 6 disruption suppresses osteoblast differentiation induced by nanotopography through the canonical Wnt signaling pathway. J Cell Physiol. 2020;235:8293‐8303. 32239701 10.1002/jcp.29674

